# Phase-specific and lifetime costs of cancer care in Ontario, Canada

**DOI:** 10.1186/s12885-016-2835-7

**Published:** 2016-10-18

**Authors:** Claire de Oliveira, Reka Pataky, Karen E. Bremner, Jagadish Rangrej, Kelvin K. W. Chan, Winson Y. Cheung, Jeffrey S. Hoch, Stuart Peacock, Murray D. Krahn

**Affiliations:** 1 Institute for Mental Health Policy Research, Centre for Addiction and Mental Health, 33 Russell Street, Room T414, Toronto, ON M5S 2S1 Canada; 2 Canadian Centre for Applied Research in Cancer Control, British Columbia Cancer Agency, 675 W 10th Ave., Vancouver, BC V5Z 1L3 Canada; 3 Toronto General Hospital, University Health Network, 200 Elizabeth Street, Room EN13-222A, Toronto, ON M5G 2C4 Canada; 4 Sunnybrook Health Sciences Centre, Institute for Clinical Evaluative Sciences, G1 72, 2075 Bayview Avenue, Toronto, ON M4N 3M5 Canada; 5 Sunnybrook Health Sciences Centre, 2075 Bayview Ave., Room T2 58, Toronto, ON M4N 3M5 Canada; 6 British Columbia Cancer Agency, 600 W. 10th Ave, Vancouver, BC V5Z 4E6 Canada; 7 University of California Davis, 2315 Stockton Blvd., Sacramento, CA 95817 USA; 8 British Columbia Cancer Agency, 675 W 10th Ave., Vancouver, BC V5Z 1L3 Canada; 9 Toronto Health Economics and Technology Assessment Collaborative, 144 College Street, Rm 600, Toronto, ON M5S 3M2 Canada

**Keywords:** Health care costs, Cancer, Neoplasms, Administrative data, Cost and cost analysis

## Abstract

**Background:**

Cancer is a major public health issue and represents a significant economic burden to health care systems worldwide. The objective of this analysis was to estimate phase-specific, 5-year and lifetime net costs for the 21 most prevalent cancer sites, and remaining tumour sites combined, in Ontario, Canada.

**Methods:**

We selected all adult patients diagnosed with a primary cancer between 1997 and 2007, with valid ICD-O site and histology codes, and who survived 30 days or more after diagnosis, from the Ontario Cancer Registry (*N* = 394,092). Patients were linked to treatment data from Cancer Care Ontario and administrative health care databases at the Institute for Clinical and Evaluative Sciences. Net costs (i.e., cost difference between patients and matched non-cancer control subjects) were estimated by phase of care and sex, and used to estimate 5-year and lifetime costs.

**Results:**

Mean net costs of care (2009 CAD) were highest in the initial (6 months post-diagnosis) and terminal (12 months pre-death) phases, and lowest in the (3 months) pre-diagnosis and continuing phases of care. Phase-specific net costs were generally lowest for melanoma and highest for brain cancer. Mean 5-year net costs varied from less than $25,000 for melanoma, thyroid and testicular cancers to more than $60,000 for multiple myeloma and leukemia. Lifetime costs ranged from less than $55,000 for lung and liver cancers to over $110,000 for leukemia, multiple myeloma, lymphoma and breast cancer.

**Conclusions:**

Costs of cancer care are substantial and vary by cancer site, phase of care and time horizon analyzed. These cost estimates are valuable to decision makers to understand the economic burden of cancer care and may be useful inputs to researchers undertaking cancer-related economic evaluations.

**Electronic supplementary material:**

The online version of this article (doi:10.1186/s12885-016-2835-7) contains supplementary material, which is available to authorized users.

## Background

Cancer is a major public health issue and represents a significant economic burden to health care systems worldwide. In Ontario, Canada’s largest province, as of January 1, 2013, 362,557 people had been diagnosed with cancer over the last 10 years (about 2.7 % of the population) [1]. The number of new cases diagnosed annually is expected to more than double from 29,649 in 1981 to 85,648 in 2016, mostly due to aging and population growth [1]. The development of new and expensive treatments has resulted in high cancer-related costs post-diagnosis, which have been increasing over time [2]. For example, for patients age 45+, mean costs nearly doubled for breast and colorectal cancers from 1997 to 2007 ($12,909 and $24,769 to $29,362 and $43,964, respectively), and increased by roughly 50 % for prostate and lung cancers for the same period (from $11,490 and $22,037 to $15,170 and $34,471, respectively) [2]. A thorough understanding of the burden of cancer care is required to ensure an optimal use of scarce health care resources. Cancer cost estimates can help inform national programs and related policies, and are an important input for economic evaluations.

Many of the seminal studies that have measured cancer costs have employed the “phase of care” approach, making it a standard method to estimate disease-related costs over time. One of its appealing aspects is that it incorporates the natural history of the disease and corresponding patterns of treatment. Furthermore, when applied to survival data, it enables the estimation of long-term costs [3, 4]. Baker and colleagues (1991) were the first to employ this method to breast and lung cancers [5]; other studies have extended this work [3, 6, 7]. One study estimated phase-specific and 5-year costs for the 18 most prevalent cancers in elderly patients in the United States [4]. The authors found that mean net costs were highest in the initial and terminal phases of care, and lowest in the continuing phase of care [4]. Most research in the United States has examined patients 65+ only; more recent work undertaken elsewhere has included patients 18+ [7, 8]. Few studies have been able to account for all relevant direct costs incurred by patients with cancer [4, 7, 8].

In Canada, medically necessary health care is funded for all permanent residents through universal public health care insurance plans managed by provincial/territorial governments. In Ontario, residents are covered by the Ontario Ministry of Health and Long-Term Care (MOHLTC). This includes services provided in hospital and by physicians as well as other services. In many cases, once care is provided outside of hospitals, patients may be required to pay out-of-pocket for direct medical costs, such as prescription drugs or home care.

The purpose of this study was to estimate the mean net costs for the 21 most prevalent cancers (and all remaining tumour sites combined) by phase of care for all patients 18+, from the perspective of the public third-party payer. In addition, it estimated 5-year and lifetime (25-year) costs for all 21 cancer sites. This study presents more comprehensive mean net costs than previous work by including, for example, costs of all physician services (including primary care) and of long-term care. Furthermore, it provides population-based cost estimates for the entire adult population over the age of 18. These estimates are lacking in the literature and will be useful to decision makers and researchers in other jurisdictions, given similar patterns of care across most developed countries.

## Methods

### Study setting

We conducted a matched cohort study to evaluate all costs incurred by the public third-party payer (MOHLTC) for patients whose first diagnosis for a primary cancer occurred between January 1, 1997 and December 31, 2007, and who survived more than 30 days after diagnosis. All costs were adjusted to 2009 Canadian dollars [9]. The study was approved by the institutional review board at Sunnybrook Health Sciences Centre, Toronto, Canada and the University of Toronto Research Ethics Board.

### Patient and control selection

Patients were identified from the Ontario Cancer Registry, a population-based cancer registry for the province of Ontario (population 13.2 million) [10]. The Ontario Cancer Registry captures approximately 95 % of all cancer diagnoses in the province of Ontario; it has been shown to be both accurate and reliable [11–14]. We included all patients 18+ assigned a single, valid International Classification of Diseases-Oncology (ICD-O) topography code corresponding to a primary cancer, and with no second cancer diagnosed within 90 days of the initial diagnosis.

We classified patients into one of the 21 most prevalent cancer sites: head and neck, esophagus, gastric, colorectal, liver, pancreas, lung, melanoma, prostate, female breast (hereafter referred to as breast), corpus uteri, cervix, ovary, bladder, renal, brain, lymphoma, multiple myeloma, leukemia, thyroid and testis. We also examined an additional category consisting of all other cancer sites combined. For each site, we selected the 20 most frequent histology codes, which were reviewed by two practising oncologists to ensure our cohort was representative of current clinical practice (see Additional file 1: Table S1).

Controls (patients without cancer) were selected from the Registered Persons Database, a population-based registry of all residents eligible for public health care insurance in Ontario. To be eligible for health care in Ontario, patients must either be a Canadian citizen, a permanent resident or among one of the newcomer groups eligible under Ontario’s Health Insurance Act; reside in the province, and be present in the province for a specified amount of time [15]. We included individuals 18+ with no cancer diagnosis before or during our analysis period and that used the health care system in the year prior to their assigned pseudo-diagnosis date.

Cases (cancer patients) and controls were matched 1:1 at two index dates – date of diagnosis and 12 months preceding the date of death (for those who died during the observation period). For the first index date, each control was randomly assigned a pseudo-diagnosis date based on the month and year of diagnosis of the matched cancer patient in our sample. For the latter index date, controls who died were matched on the same date of death as the cancer patient. To match each case to a control, we calculated a propensity score of having cancer, through the use of a logit model, using age, sex, neighbourhood income quintile at the Census tract level [16], “rural and small town” indicator from Statistics Canada [17], comorbidity, measured by the Johns Hopkins aggregated diagnosis groups (excluding aggregated diagnosis group 32 – malignancy) [18] in the year prior to the index date, and residence in a long-term care facility at index date. We selected the closest control that met the following criteria: age +/− 2 years at the index date; same sex (hard match); and a propensity score within a caliper width of 0.05 standard deviations [19]. We were able to find a suitable control for 98 % (*N* = 393,154) of our initial cohort (*N* = 402,399).

### Data sources

**Fig. 1:**
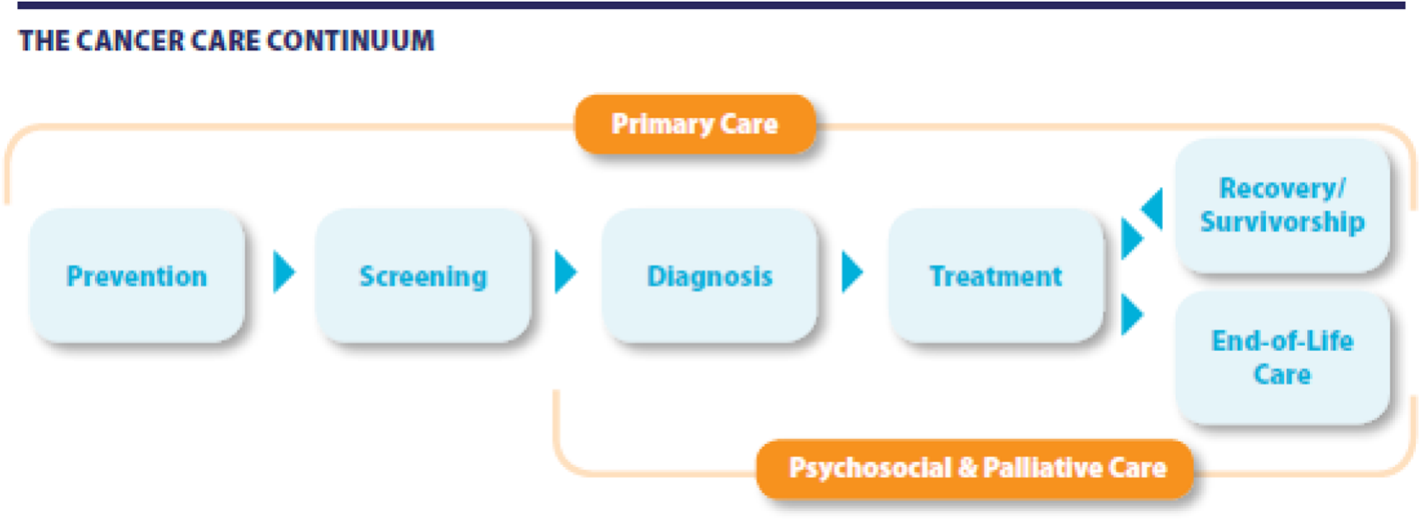
Cancer care continuum in Ontario. Source: Ontario Cancer Plan IV 2015-2019, Cancer Care Ontario https://cancercare.on.ca/common/pages/UserFile.aspx?fileId=333871

We obtained data on all patients from pre-diagnosis to diagnosis and treatment to recovery/survivorship and/or end-of-life care (Fig. 1). Cancer-specific treatment data (chemotherapy and radiation therapy data) were obtained from Cancer Care Ontario, the provincial agency responsible for improving cancer services in Ontario. Data on all other resources from pre-diagnosis to recovery/survivorship and/or end-of-life care were obtained from the Institute for Clinical Evaluative Sciences in Toronto, Ontario. The combined set of databases included: New Drug Funding Program (chemotherapy), Activity Level Reporting System (radiation therapy), Ontario Health Insurance Plan claims database (all physician services, including primary care physicians, specialists and other physicians, and diagnostic tests and laboratory services), Ontario Drug Benefit program database (outpatient prescription drugs for patients age 65+ and/or on social assistance only), Canadian Institute for Health Information-Discharge Abstract Database (inpatient hospitalizations), Canadian Institute for Health Information-National Ambulatory Care Reporting System (ambulatory care, which includes same-day surgeries/procedures and emergency department visits), Continuing Care Reporting System (other institution-based care), Ontario Home Care Administrative System and Home Care Database (home care). (See Additional file 1: Table S2 for a detailed description.) These databases have been described and validated in the literature; the collection and reporting of the data by hospitals and other health care institutions follow the Ontario Healthcare Reporting Standards/Management Information Systems [20]. Furthermore, these databases have been used in previous work as a source of data for costing analyses in Ontario [2, 21, 22]. They include the cost of the vast majority of health care resources covered under the Ontario public health care insurance plan (roughly 90 %) [20]; however, they do not cover costs with community service agencies. In addition, they do not capture costs covered under private health care plans, such as costs with outpatient prescription drugs for patients under the age of 65, and other health care costs paid out-of-pocket. All datasets were linked through the use of unique encoded identifiers and analyzed at the Institute for Clinical Evaluative Sciences.

Cost estimates for inpatient hospitalizations, same-day surgeries/procedures and emergency department visits were obtained by multiplying the resource intensity weight (measure of resource utilization intensity) by the cost per weighted case (unit cost) [20, 23–25]. Cost estimates for other resources were either available in the data or obtained from other sources [20, 26]. The costing methods followed the guidelines of the Canadian Agency for Drugs and Technology in Health [27] and were based on previous cancer costing work done in Ontario [2, 21, 22].

### Study design and analysis

#### Phase-specific net costs of care

All analyses were done using SAS ® version 9.2. We used a phase-based approach [4, 5, 28, 29] to estimate costs incurred before and after diagnosis, and to account for differences in follow up time. All patients had a pre-diagnosis phase, which we defined as the 3 months before diagnosis. This phase typically includes diagnostic testing to establish the cancer diagnosis [30, 31]. We divided the time between diagnosis and death into three clinically relevant phases of care: 1) initial, which includes the primary course of therapy and any adjuvant therapy, and defined as the 6 months after diagnosis (including date of diagnosis); 2) continuing, which encompasses ongoing surveillance and active follow-up treatment for cancer recurrence and/or new primary cancers, and expressed as an annual estimate; and 3) terminal, which captures the intensive services, often palliative in nature, provided in the 12 months before death. The lengths of the phases were based on clinical knowledge of the disease and joinpoint analysis [4, 5, 32]. We employed the same length across all sites to ensure comparability. Patients who died had their observation time, up to 12 months, first assigned to the terminal phase; any remaining time, as well as all time of patients who survived, was then assigned to the initial phase and finally to the continuing phase [29].

We employed the ‘net cost’ method [28, 29] to obtain an estimate of the cost attributable to cancer. This method consists of subtracting the costs incurred by patients from those incurred by matched controls. The mean net cost (C) for each phase of care and cancer site was defined as C _phase_ = C ^P^_phase_ - C ^C^_phase_, where C denotes mean net cost, and subscripts P and C denote patient and control subjects, respectively. The corresponding variance was defined as Var (C _phase_) = Var (C ^P^_phase_) + Var (C ^C^_phase_).^1^ Mean net costs were estimated for each cancer site, sex and phase of care. We also calculated confidence intervals (CIs) for each cost estimate through Taylor series expansion based on asymptotic assumptions [33]. Costs by resource were also estimated and are available upon request.

#### Mean 1- and 5-year net costs of care

We estimated mean undiscounted 1- and 5-year net costs (C1Y and C5Y, respectively) by applying monthly survival probabilities, obtained from Cancer Care Ontario, to the mean monthly net cost estimates for patients in the initial, continuing, and terminal phases described above. Mean 1- and 5-year net costs, respectively, were calculated as follows, where Init_i_, Cont_i_, Term_i_ represent the amount of time each patient spent in month *i* in the initial, continuing and terminal phases, respectively; C_init_, C_cont_ and C_term_ represent the phase-specific net cost, and P_i_^AD^ represents the probability of dying of any cause (cancer- or non-cancer-related) in month *i* [4]^2^:

$$ \begin{array}{l}\mathrm{C}1\mathrm{Y} = {\mathrm{C}}_{\mathrm{i}\mathrm{n}\mathrm{i}\mathrm{t}}*{\displaystyle {\sum}_{\mathrm{i}}}\left({{\mathrm{P}}_{\mathrm{i}}}^{\mathrm{AD}}*\ \mathrm{I}\mathrm{n}\mathrm{i}{\mathrm{t}}_{\mathrm{i}}\right) + {\mathrm{C}}_{\mathrm{cont}}*{\displaystyle {\sum}_{\mathrm{i}}}\left({{\mathrm{P}}_{\mathrm{i}}}^{\mathrm{AD}}*\ \mathrm{C}\mathrm{on}{\mathrm{t}}_{\mathrm{i}}\right) + {\mathrm{C}}_{\mathrm{t}\mathrm{e}\mathrm{r}\mathrm{m}}*{\displaystyle {\sum}_{\mathrm{i}}}\left({{\mathrm{P}}_{\mathrm{i}}}^{\mathrm{AD}}*\ \mathrm{T}\mathrm{e}\mathrm{r}{\mathrm{m}}_{\mathrm{i}}\right)\\ {} + \left(1\ \hbox{-} {\displaystyle {\sum}_{\mathrm{i}}}{{\mathrm{P}}_{24}}^{\mathrm{AD}}\right)\ *\ \left(6*{\mathrm{C}}_{\mathrm{i}\mathrm{n}\mathrm{i}\mathrm{t}} + 6*{\mathrm{C}}_{\mathrm{cont}}\right),\mathrm{where}\kern0.24em \mathrm{i} = \left\{1, \dots,\ 23\right\};\end{array} $$

$$ \begin{array}{l}\mathrm{C}5\mathrm{Y} = {\mathrm{C}}_{\mathrm{i}\mathrm{n}\mathrm{i}\mathrm{t}}*{\displaystyle {\sum}_{\mathrm{i}}}\left({{\mathrm{P}}_{\mathrm{i}}}^{\mathrm{AD}}*\ \mathrm{I}\mathrm{n}\mathrm{i}{\mathrm{t}}_{\mathrm{i}}\right) + {\mathrm{C}}_{\mathrm{cont}}*{\displaystyle {\sum}_{\mathrm{i}}}\left({{\mathrm{P}}_{\mathrm{i}}}^{\mathrm{AD}}*\ \mathrm{C}\mathrm{on}{\mathrm{t}}_{\mathrm{i}}\right) + {\mathrm{C}}_{\mathrm{t}\mathrm{e}\mathrm{r}\mathrm{m}}*{\displaystyle {\sum}_{\mathrm{i}}}\left({{\mathrm{P}}_{\mathrm{i}}}^{\mathrm{AD}}*\ \mathrm{T}\mathrm{e}\mathrm{r}{\mathrm{m}}_{\mathrm{i}}\right)\\ {} + \left(1\ \hbox{-} {\displaystyle {\sum}_{\mathrm{i}}}{{\mathrm{P}}_{72}}^{\mathrm{AD}}\right)\ *\ \left(6*{\mathrm{C}}_{\mathrm{i}\mathrm{n}\mathrm{i}\mathrm{t}} + 54*{\mathrm{C}}_{\mathrm{cont}}\right),\mathrm{where}\ \mathrm{i} = \left\{1, \dots,\ 71\right\}.\end{array} $$

To estimate 1-year net costs, we used monthly survival probabilities for 24 months as patients who died in the second year after diagnosis (13 ≤ month *i* < 24) would have been in their last year of life for some portion of the first year, thus incurring terminal costs in both time periods. We applied the same rule to estimate undiscounted 5-year net costs, in line with previous work [4]. We also estimated mean discounted 5-year net costs using a 5 % discount rate annually [18]. We calculated 95 % CIs for each estimate.

#### Mean lifetime net costs of care

We combined phase-specific cost estimates with long-term survival curves to calculate undiscounted and discounted lifetime costs from diagnosis to death, in line with previous research [29]. This was done by taking a weighted average of estimated cancer-related costs for patients surviving different lengths of time, up to 25 years after diagnosis. We calculated 95 % CIs for each estimate. One of the limitations of this approach is that long-term survival tends to have lower continuing care costs than short-term survivals [29].

## Results

### Patients

Table 1 describes the characteristics of the cohort of matched cancer patients (*N* = 394,092). The majority had breast, prostate, colorectal and lung cancers (≈60 % combined). Patients had a mean age of 63 years; 51 % were male. They were fairly equally distributed across neighbourhood income quintiles and lived mostly in urban areas (85 %); few lived in long-term facilities (1 %) at diagnosis. Approximately half required speciality care (55 %) (i.e. an unstable chronic condition) in the year prior to diagnosis. Most patients were diagnosed in the later years of our analysis period.

**Table 1:** Characteristics of patients diagnosed with cancer

Characteristic	Number	Percent
Overall sample	394,092	100.0
Type of cancer		
Breast (female)	68,147	17.3
Prostate	67,539	17.1
Colorectal	56,635	14.4
Lung	41,378	10.5
Melanoma	16,892	4.3
Head and neck	12,291	3.1
Corpus uteri	12,109	3.1
Bladder	12,048	3.1
Thyroid	11,339	2.9
Lymphoma	10,246	2.6
Renal	9861	2.5
Leukemia	7897	2.0
Gastric	7889	2.0
Ovary	6999	1.8
Pancreas	6231	1.6
Brain	5358	1.4
Cervix	4753	1.2
Esophagus	4261	1.1
Myeloma	4214	1.1
Testis	3015	0.8
Liver	2580	0.7
Other tumours	22,410	5.7
Age in years at diagnosis		
Mean (SD)	63.4 (13.9)	--
Median (IQR)	65 (54–74)	--
Sex		
Male	201,050	51.0
Female	193,042	49.0
Neighbourhood income quintile		
1 – Low	74,465	18.9
2 – Medium-low	80,541	20.4
3 – Medium	78044	19.8
4 – Medium-High	78,250	19.9
5 - High	82,792	21.0
Urban/rural residence		
Urban	336,275	85.3
Rural	57,817	14.7
Residence in long-term care facility	3972	1.0
Collapsed Ambulatory Diagnostic Group		
Acute Minor	39,667	10.1
Acute Major	124,683	31.6
Likely to recur	58,043	14.7
Asthma	2327	0.6
Chronic, unstable	217,622	55.2
Chronic, stable	75,512	19.2
Specialty, unstable	6304	1.6
Specialty, stable	2488	0.6
Eye, dental	9576	2.4
Psychosocial	12,616	3.2
Prevention	39,324	1.0
Pregnancy	1237	0.3
Year of diagnosis		
1997	30,839	7.8
1998	31,230	7.9
1999	32,437	8.2
2000	33,792	8.6
2001	35,222	8.9
2002	35,911	9.1
2003	35,724	9.1
2004	37,848	9.6
2005	39,423	10.0
2006	40,374	10.2
2007	41,292	10.5

*SD* Standard deviation, *IQR* inter-quartile range

Data sources: Ontario Cancer Registry, Canada Census data, Statistics Canada Postal Code Conversion File and administrative health care data housed at the Institute for Clinical Evaluative Sciences

Controls were fairly well matched to cases. The quality of the match was generally better for cases matched at diagnosis than those matched 12 month before death, and for cancer sites with larger numbers of patients (not shown; results can be found in the Additional files 2 and 3).

### Phase-specific net costs of care

Mean net costs were highest in the initial and terminal care phases, and lowest in the pre-diagnosis and continuing care phases, following a u-shaped curve from diagnosis to death (Table 2; Additional file 1: Figure S1). For the 3-month pre-diagnosis phase, net costs were lowest for bladder ($236 and $217, for men and women respectively) and esophagus for women only ($221). Net costs for this phase were highest for liver ($3381 and $2893 for men and women, respectively) and multiple myeloma ($3142 and $2609 for men and women, respectively). High pre-diagnosis costs were mainly due to diagnostic testing and hospital admissions.

**Table 2:** Mean net costs of care by phase of care and tumour site^a^

Tumour Site	Phase, estimated cost (95 % CI)			
	Pre-diagnosis (3 months)	Initial (6 months)	Continuing (annual)	Terminal (12 months)
*Males*				
Head and neck	$595 ($326–$865)	$19,702 ($19,691–$19,714)	$5151 ($5143–$5159)	$37,346 ($37,332–$37,360)
Esophagus	$818 ($455–$1180)	$41,567 ($41,539–$41,596)	$5491 ($5474–$5509)	$54,354 ($54,336–$54,371)
Gastric	$848 ($481–$1215)	$32,240 ($32,203–$32,278)	$3329 ($3315–$3342)	$53,708 ($53,695–$53,722)
Colorectal	$275 (−$101-$651)	$25,138 ($25,131–$25,146)	$5446 ($5442–$5451)	$32,408 ($32,401–$32,415)
Liver	$3381 ($2906–$3855)	$21,355 ($21,325–$21,384)	$11,954 ($11,937–$11,971)	$30,265 ($30,242–$30,289)
Pancreas	$1892 ($1468–$2315)	$29,979 ($29,950–$30,008)	$6296 ($6272–$6319)	$54,152 ($54,138–$54,167)
Lung	$1833 ($1458–$2209)	$22,409 ($22,402–$22,417)	$5533 ($5526–$5539)	$39,241 ($39,236–$39,247)
Melanoma	$553 ($331–$774)	$4649 ($4635–$4664)	$4005 ($3998–$4012)	$18,494 ($18,479–$18,509)
Prostate	$637 ($375–$899)	$8394 ($8391–$8397)	$5017 ($5015–$5020)	$17,391 ($17,385–$17,397)
Bladder	$236 (−$189–$661)	$10,429 ($10,412–$10,447)	$3394 ($3386–$3403)	$35,749 ($35,737–$35,760)
Renal	$1503 ($1111–$1895)	$14,950 ($14,936–$14,964)	$3991 ($3981–$4002)	$38,292 ($38,274–$38,309)
Brain	$1548 ($1192–$1904)	$33,241 ($33,227–$33,225)	$6563 ($6546–$6581)	$72,463 ($72,444–$72,483)
Lymphoma	$1484 ($1125–$1843)	$17,831 ($17,820–$17,842)	$6276 ($6268–$6285)	$59,202 ($59,182–$59,222)
Myeloma	$3142 ($2675–$3609)	$24,447 ($24,418–$24,476)	$15,153 ($15,138–$15,169)	$43,989 ($43,969–$44,010)
Leukemia	$1325 ($1006–$1645)	$18,214 ($18,194–$18,233)	$8035 ($8024–$8045)	$74,857 ($74,837–$74,877)
Thyroid	$1020 ($757–$1282)	$9837 ($9828–$9846)	$3382 ($3372–$3391)	$33,459 ($33,408–$33,511)
Testis	$1325 ($1106–$1544)	$11,201 ($11,190–$11,211)	$2264 ($2255–$2273)	$77,814 ($77,721–$77,907)
All other tumour sites^b^	$1469 ($1075–$1862)	$18,730 ($18,720–$18,740)	$5878 ($5870–$5886)	$42,047 ($42,037–$42,057)
*Females*				
Head and neck	$1217 ($877–$1557)	$20,242 ($20,212–$20,271)	$7049 ($7032–$7065)	$36,382 ($36,361–$36,402)
Esophagus	$221 (−$218–$660)	$42,658 ($42,633–$42,684)	$6744 ($6703–$6785)	$51,728 ($51,699–$51,757)
Gastric	$681 ($214–$1149)	$29,940 ($29,922–$29,958)	$2660 ($2634–$2685)	$52,551 ($52,533–$52,570)
Colorectal	$542 ($122–$963)	$24,765 ($24,753–$24,777)	$5349 ($5343–$5355)	$31,120 ($31,113–$31,127)
Liver	$2893 ($2441–$3346)	$19,331 ($19,252–$19,411)	$7764 ($7707–$7821)	$27,850 ($27,813–$27,888)
Pancreas	$1716 ($1282–$2150)	$31,953 ($31,924–$31,981)	$8734 ($8702–$8767)	$53,320 ($53,303–$53,337)
Lung	$2047 ($1648–$2445)	$21,583 ($21,571–$21,596)	$6251 ($6243–$6260)	$35,664 ($35,657–$35,671)
Melanoma	$437 ($236–$638)	$4110 ($4097–$4122)	$3872 ($3864–$3880)	$16,115 ($16,095–$16,134)
Female breast	$1216 ($944–$1487)	$12,219 ($12,213–$12,224)	$6741 ($6738–$6744)	$18,593 ($18,587–$18,598)
Corpus uteri	$852 ($558–$1145)	$12,083 ($12,073–$12,093)	$3320 ($3312–$3327)	$22,577 ($22,560–$22,593)
Cervix	$781 ($554–$1007)	$14,448 ($14,442–$14,454)	$2833 ($2823–$2842)	$31,796 ($31,774–$31,819)
Ovary	$1490 ($1155–$1825)	$22,532 ($22,518–$22,546)	$4100 ($4089–$4110)	$34,670 ($34,657–$34,684)
Bladder	$217 (−$222–$655)	$10,886 ($10,850–$10,923)	$5127 ($5109–$5145)	$37,087 ($37,069–$37,105)
Renal	$2335 ($1883–$2787)	$15,602 ($15,573–$15,632)	$4525 ($4511–$4539)	$40,810 ($40,787–$40,834)
Brain	$2004 ($1619–$2389)	$30,683 ($30,669–$30,697)	$9883 ($9854–$9912)	$81,385 ($81,360–$81,411)
Lymphoma	$1838 ($1488–$2187)	$16,885 ($16,860–$16,910)	$6274 ($6263–$6285)	$43,600 ($43,579–$43,621)
Myeloma	$2609 ($2077–$3140)	$24,052 ($24,012–$24,092)	$15,255 ($15,238–$15,272)	$45,871 ($45,849–$45,892)
Leukemia	$706 ($301–$1112)	$24,256 ($24,236–$24,276)	$9949 ($9933–$9965)	$69,531 ($69,507–$69,556)
Thyroid	$946 ($725–$1167)	$9098 ($9093–$9102)	$3396 ($3390–$3402)	$28,704 ($28,654–$28,754)
All other tumour sites^b^	$1455 ($1059–$1850)	$18,288 ($18,271–$18,305)	$5790 ($5780–$5800)	$43,214 ($43,202–$43,226)

^a^The initial phase of care is the first 6 months following diagnosis, the terminal phase is the final 12 months of life, and the continuing phase is all the months between the initial and last year of life phases. Net costs in the continuing phase of care are an annual estimate. Net costs in the last year of life combine the cost for cancer patients dying of cancer and those dying of other causes. All estimates are in 2009 dollars

^b^All other tumour sites includes salivary gland, small intestine, appendix, intrahepatic bile duct, gallbladder and extrahepatic bile ducts, unspecified digestive organs, pleura, thymus, heart, mediastinum, other respiratory organs, bones and joints, reticulo-endothelial, spleen, connective tissue/nerves, retroperitoneum and peritoneum, soft tissue, breast (male only), labia and clitoris, vulva, vagina, other female genitals, placenta, penis, epididymis, spermatic cord, scrotum, other and unspecified male genitals, other urinary organs, ureter, eye, orbit and lacrimal gland, eye (unspecified), cerebral and spinal meninges, meninges NOS, spinal cord, cranial nerves, other nervous system, adrenal glands, parathyroid gland, pituitary gland, craniopharyngeal duct, pineal gland, other endocrine glands and miscellaneous (ill-defined and unknown organs)

Data sources: Cancer Care Ontario and administrative health data housed at the Institute for Clinical Evaluative Sciences

Net costs increased greatly in the 6-month initial phase and were highest for esophageal, brain, pancreas and gastric cancers. Costs for these sites were greater than $29,000, with the highest cost for esophageal cancer ($41,567 and $42,658 for men and women, respectively). Net costs were lowest for melanoma ($4649 and $4110 for men and women, respectively). Costs were mainly due to hospital admissions and, to a lesser extent, physician services.

Net costs decreased for the continuing phase and were highest for multiple myeloma ($15,153 and $15,255 for men and women, respectively), and lowest for testicular cancer for men ($2264) and gastric cancer for women ($2660). Hospital admissions and other institution-based care made up the bulk of the total cost.

Net costs were highest in the 12-month terminal phase of care. These were greater than $70,000 for patients with brain ($72,463 and $81,385 for men and women, respectively) and testicular cancers ($77,814), and lowest for melanoma ($18,494 and $16,115 for men and women, respectively), prostate ($17,391) and breast ($18,593) cancers. Again, the main drivers of costs were hospital admissions and, to a lesser extent, home and other institution-based care.

For most cancer sites and phases (except the pre-diagnosis phase), CIs did not overlap among males and females, suggesting cost differences by sex. The exceptions were esophageal cancer in the initial phase, and lymphoma, colorectal and thyroid cancers in the continuing phase, indicating similarity in costs. There was no clear pattern in the ranking of costs by cancer site between sexes. Hospitalizations comprised the largest portion of net costs across all post-diagnosis phases (not shown; results available upon request).

### Mean 1-year, 5-year and lifetime net costs of care

The proportion of patients alive 1 year after diagnosis was greater than 95 % for testicular, thyroid, prostate and breast cancers, and melanoma, but only 30 % for patients with pancreatic cancer (Table 3). Undiscounted mean 1-year net costs were lowest for melanoma, thyroid and prostate cancers, and highest for esophageal cancer. One-year net costs accounted for roughly 80 % of the undiscounted 5-year net cost for esophageal and pancreatic cancers.

**Table 3:** Mean 5-year net costs of care by tumour site*

Tumour Site	% alive after diagnosis		Undiscounted costs, $ (95 % CI)		5-year discounted costs at 5 %, $ (95 % CI)
	Year 1	Year 5	Year 1	Year 5	
*Males*					
Head and neck	82.4	56.9	$25,127 ($25,112–$25,141)	$44,305 ($44,270–$44,340)	$42,336 ($42,303–$42,369)
Esophagus	43.0	13.4	$38,833 ($38,814–$38,852)	$49,260 ($49,230–$49,291)	$48,348 ($48,319–$48,378)
Gastric	50.9	20.3	$33,633 ($33,611–$33,654)	$44,852 ($44,819–$44,885)	$43,887 ($43,855–$43,919)
Colorectal	84.1	55.8	$27,149 ($27,140–$27,158)	$46,892 ($46,872–$46,913)	$44,874 ($44,855–$44,893)
Liver	54.5	25.0	$21,044 ($21,022–$21,067)	$35,020 ($34,981–$35,059)	$33,680 ($33,643–$33,718)
Pancreas	29.5	7.7	$28,067 ($28,054–$28,080)	$34,181 ($34,161–$34,202)	$33,661 ($33,641–$33,681)
Lung	43.1	15.3	$22,468 ($22,463–$22,473)	$29,788 ($29,780–$29,797)	$29,150 ($29,142–$29,159)
Melanoma	94.0	75.5	$8171 ($8153–$8188)	$23,022 ($22,981–$23,063)	$21,440 ($21,401–$21,478)
Prostate	96.9	83.9	$11,267 ($11,262–$11,271)	$30,322 ($30,308–$30,336)	$28,219 ($28,206–$28,232)
Bladder	84.6	58.2	$16,678 ($16,660–$16,697)	$31,776 ($31,736–$31,815)	$30,223 ($30,186–$30,260)
Renal	83.6	65.8	$20,613 ($20,597–$20,629)	$33,853 ($33,817–$33,890)	$32,500 ($32,466–$32,535)
Brain	49.2	20.1	$29,142 ($29,131–$29,154)	$39,489 ($39,460–$39,518)	$38,370 ($38,343–$38,397)
Lymphoma	84.7	66.2	$25,830 ($25,815–$25,845)	$50,085 ($50,048–$50,122)	$47,540 ($47,506–$47,574)
Myeloma	79.5	40.1	$31,938 ($31,908–$31,967)	$68,056 ($67,997–$68,115)	$64,414 ($64,358–$64,470)
Leukemia	79.3	58.2	$30,642 ($30,622–$30,662)	$59,335 ($59,292–$59,378)	$56,420 ($56,380–$56,461)
Thyroid	96.7	91.6	$12,153 ($12,138–$12,168)	$26,361 ($26,309–$26,413)	$24,789 ($24,742–$24,837)
Testis	98.6	96.2	$14,010 ($13,993–$14,027)	$24,049 ($23,996–$24,103)	$22,919 ($22,870–$22,968)
All other tumour sites	72.6	47.4	$22,525 ($22,515–$22,535)	$38,459 ($38,436–$38,482)	$36,868 ($36,846–$36,890)
*Females*					
Head and neck	80.8	57.9	$25,254 ($25,222–$25,285)	$47,882 ($47,811–$47,953)	$45,474 ($45,407–$45,540)
Esophagus	41.6	16.3	$37,896 ($37,870–$37,922)	$47,490 ($47,440–$47,540)	$46,638 ($46,590–$46,685)
Gastric	49.9	24.5	$31,748 ($31,731–$31,766)	$41,482 ($41,442–$41,522)	$40,601 ($40,563–$40,639)
Colorectal	83.7	58.1	$25,849 ($25,837–$25,861)	$44,187 ($44,160–$44,213)	$42,303 ($42,278–$42,328)
Liver	54.5	21.1	$18,394 ($18,340–$18,448)	$29,933 ($29,829–$30,036)	$28,846 ($28,747–$28,944)
Pancreas	29.9	7.6	$28,940 ($28,925–$28,956)	$35,610 ($35,584–$35,635)	$35,017 ($34,992–$35,041)
Lung	50.7	21.6	$21,909 ($21,901–$21,918)	$31,010 ($30,996–$31,025)	$30,184 ($30,170–$30,197)
Melanoma	96.1	84.8	$6717 ($6701–$6734)	$21,533 ($21,487–$21,578)	$19,907 ($19,865–$19,949)
Female breast	96.6	81.8	$15,752 ($15,745–$15,758)	$40,543 ($40,526–$40,560)	$37,821 ($37,805–$37,837)
Corpus uteri	94.5	81.8	$14,284 ($14,270–$14,298)	$27,818 ($27,777–$27,859)	$26,345 ($26,307–$26,383)
Cervix	90.5	73.3	$18,160 ($18,148–$18,172)	$30,815 ($30,774–$30,857)	$29,495 ($29,457–$29,533)
Ovary	82.1	45.5	$25,740 ($25,723–$25,757)	$42,352 ($42,314–$42,391)	$40,734 ($40,698–$40,770)
Bladder	77.5	52.5	$17,567 ($17,533–$17,602)	$34,625 ($34,552–$34,698)	$32,864 ($32,795–$32,932)
Renal	85.5	70.2	$21,281 ($21,253–$21,310)	$36,096 ($36,038–$36,154)	$34,546 ($34,491–$34,601)
Brain	48.7	24.9	$32,686 ($32,672–$32,700)	$45,533 ($45,500–$45,566)	$44,293 ($44,262–$44,323)
Lymphoma	87.4	70.5	$21,451 ($21,425–$21,477)	$43,729 ($43,671–$43,787)	$41,338 ($41,283–$41,392)
Myeloma	79.9	40.0	$31,650 ($31,613–$31,686)	$68,302 ($68,233–$68,371)	$64,672 ($64,606–$64,737)
Leukemia	78.1	58.2	$32,326 ($32,304–$32,348)	$61,659 ($61,603–$61,715)	$58,597 ($58,544–$58,649)
Thyroid	98.9	97.2	$10,976 ($10,968–$10,984)	$24,644 ($24,610–$24,677)	$23,100 ($23,070–$23,130)
All other tumour sites	70.9	47.6	$22,558 ($22,543–$22,573)	$38,678 ($38,645–$38,710)	$37,056 ($37,025–$37,087)

* Phase-specific net cost of care estimates were applied to 5-year survival probabilities among cancer patients diagnosed 1997–2007. All cost estimates are in 2009 dollars

Data sources: Ontario Cancer Registry data (survival), and Cancer Care Ontario and administrative health data housed at the Institute for Clinical Evaluative Sciences (mean net costs by phase of care)

**Fig. 2:**
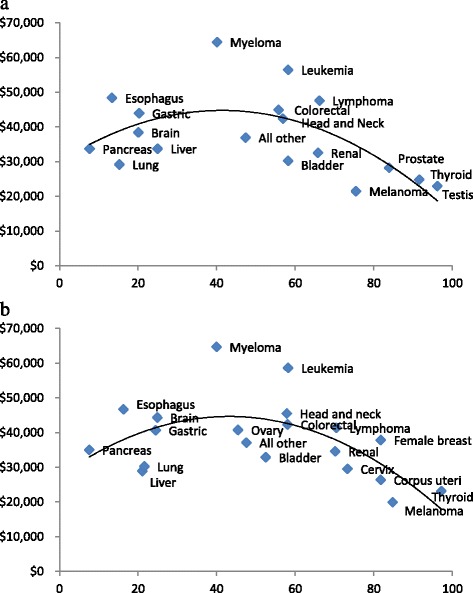
Association between discounted 5-year net costs by percentage of patients alive 5 years after diagnosis for males **a** and females **b** (trendline is a polynomial of order 2)

The proportion of patients alive 5 years after diagnosis was greater than 90 % for testicular and thyroid cancers only, and less than 20 % for those with esophageal and pancreatic cancers (Table 3). Undiscounted mean 5-year net costs varied quite a bit across cancer sites, from less than $25,000 for melanoma, thyroid and testicular cancers to more than $55,000 for multiple myeloma and leukemia. The same findings held for discounted mean 5-year net costs. In addition, we mapped the association between discounted 5-year net costs by the percentage of patients alive 5 years after diagnosis for males and females. We found that costs followed an inverted U-shaped curve with survival (Fig. 2).

Discounted lifetime net costs ranged from less than $50,000, for melanoma, liver (females only), testicular and lung cancers, to over $95,000, for leukemia and multiple myeloma (Table 4).

**Table 4:** Mean lifetime (25-year) net costs of care by tumour site*

Tumour Site	Undiscounted costs, $ (95 % CI)	Discounted costs at 5 %, $ (95 % CI)
*Males*		
Head and neck	$83,700 ($83,627–$83,774)	$66,249 ($66,194–$66,305)
Esophagus	$77,260 ($77,210–$77,311)	$69,048 ($69,005–$69,092)
Gastric	$73,490 ($73,435–$73,545)	$64,624 ($64,578–$64,670)
Colorectal	$86,536 ($86,494–$86,579)	$68,686 ($68,654–$68,718)
Liver	$60,501 ($60,435–$60,567)	$51,587 ($51,531–$51,643)
Pancreas	$65,495 ($65,458–$65,531)	$57,666 ($57,635–$57,697)
Lung	$52,356 ($52,341–$52,371)	$46,041 ($46,029–$46,054)
Melanoma	$60,591 ($60,490–$60,693)	$42,224 ($42,153–$42,296)
Prostate	$79,147 ($79,110–$79,184)	$56,156 ($56,129–$56,182)
Bladder	$64,729 ($64,646–$64,811)	$50,036 ($49,973–$50,098)
Renal	$68,757 ($68,673–$68,840)	$53,992 ($53,930–$54,054)
Brain	$100,364 ($100,287–$100,441)	$88,322 ($88,258–$88,386)
Lymphoma	$114,574 ($114,484–$114,665)	$85,723 ($85,657–$85,788)
Myeloma	$118,255 ($118,156–$118,354)	$96,847 ($96,766–$96,929)
Leukemia	$128,641 ($128,548–$128,734)	$99,506 ($99,436–$99,576)
Thyroid	$84,113 ($83,918–$84,308)	$54,921 ($54,799–$55,043)
Testis	$75,458 ($75,238–$75,678)	$48,550 ($48,417–$48,683)
All other tumour sites	$76,634 ($76,584–$76,683)	$61,520 ($61,483–$61,558)
*Females*		
Head and neck	$95,704 ($95,558–$95,850)	$74,332 ($74,220–$74,443)
Esophagus	$76,240 ($76,148–$76,332)	$67,714 ($67,638–$67,790)
Gastric	$70,738 ($70,654–$70,821)	$61,429 ($61,364–$61,494)
Colorectal	$83,535 ($83,480–$83,591)	$65,944 ($65,902–$65,986)
Liver	$50,349 ($50,175–$50,523)	$43,221 ($43,076–$43,366)
Pancreas	$67,424 ($67,377–$67,472)	$59,192 ($59,152–$59,232)
Lung	$52,940 ($52,916–$52,964)	$46,198 ($46,177–$46,218)
Melanoma	$70,249 ($70,108–$70,390)	$45,730 ($45,637–$45,823)
Female breast	$110,346 ($110,300–$110,392)	$76,113 ($76,081–$76,144)
Corpus uteri	$70,587 ($70,471–$70,703)	$49,841 ($49,762–$49,921)
Cervix	$69,230 ($69,094–$69,367)	$50,208 ($50,119–$50,296)
Ovary	$70,831 ($70,754–$70,908)	$58,433 ($58,374–$58,492)
Bladder	$75,617 ($75,459–$75,775)	$57,430 ($57,312–$57,548)
Renal	$76,638 ($76,511–$76,766)	$59,183 ($59,088–$59,279)
Brain	$107,188 ($107,101–$107,274)	$80,728 ($80,672–$80,784)
Lymphoma	$107,514 ($107,371–$107,656)	$77,860 ($77,758–$77,961)
Myeloma	$119,958 ($119,845–$120,072)	$97,988 ($97,894–$98,082)
Leukemia	$138,749 ($138,617–$138,881)	$106,112 ($106,015–$106,208)
Thyroid	$86,781 ($86,633–$86,929)	$54,625 ($54,537–$54,714)
All other tumour sites	$80,820 ($80,748–$80,891)	$63,505 ($63,451–$63,558)

* Phase-specific net cost of care estimates were applied to 25-year survival probabilities among cancer patients diagnosed 1997–2007. All cost estimates are in 2009 dollars

Data sources: Ontario Cancer Registry data (survival), and Cancer Care Ontario and administrative health data housed at the Institute for Clinical Evaluative Sciences (mean net costs by phase of care)

## Discussion

We used administrative health care data to estimate phase-specific, 5-year and lifetime net costs for the 21 most prevalent cancers individually and all other cancer sites combined. Our findings suggest that cancer-related costs are substantial and vary by cancer site, phase of care and time horizon of analysis. We found that net costs followed a U-shaped curve consistent with previous research ([4, 7, 8, 21] de Oliveira C, Pataky R, Bremner K, et al. Estimating the cost of cancer care in British Columbia and Ontario: a Canadian inter-provincial comparison, submitted), where costs were higher in the initial and terminal phases, and lower in the pre-diagnosis and continuing phases. Five-year and lifetime costs were generally highest among patients diagnosed with hematological cancers.

Disease-specific estimates of costs are of great importance in the health economics and health policy fields [34]. These estimates can be used to help justify screening and intervention programs, provide a foundation for policy and planning relative to prevention and control initiatives, and assist in the allocation of research funds to specific diseases. Furthermore, phase-specific cost estimates constitute an important input for economic evaluations, in particular those designed to evaluate prevention and screening interventions.

Our findings are largely in accordance with previous work using SEER-Medicare data in the United States [4]. Yabroff et al. (2008) also found that net costs in the initial phase were highest for cancers with low survival, such as brain, pancreas, esophageal and gastric cancers, and lowest for cancers with high survival, such as melanoma and prostate cancer [4]. These findings are also in line with other research examining patients 18+ [8].

The ranking of our mean discounted 5-year net costs was similar to that found in the SEER-Medicare population as well. Previous research found high 5-year costs for esophageal cancer and lymphoma, and low 5-year costs for melanoma [4]. Furthermore, we found that cancers with 5-year relative survival rates between roughly 40 and 66 % tended to have the highest mean net costs, similar to findings from New Zealand [8]. As suggested by Blakely et al. (2015), the idea is that patients with cancers with poor prognosis, such pancreatic cancer, as well as those with cancers with good prognosis, such as melanoma and thyroid cancer, do not consume high costs, as the former do not live long while the latter are able to respond more fully to initial treatment [8]. Patients with the highest 5-year net costs are those with average prognosis cancers, such as multiple myeloma and leukemia, who consume more resources due to recurrences and available treatments that are able to extend survival [8].

We found lifetime net costs were highest among hematological cancers, such as leukemia, multiple myeloma and lymphoma, and breast cancer. Few studies have estimated lifetime costs for all cancer sites; most have examined either colorectal [29, 34, 35] or prostate cancers [36] only. Our lifetime cost for colorectal cancer was higher than the SEER-Medicare estimate. This difference, in addition to the high lifetime costs for breast cancer and leukemia, is likely due to the inclusion of younger patients in our sample. The SEER-Medicare data include patients age 65+ only. Cost estimates using the SEER-Medicare data do not include the higher costs for younger cancer patients who are typically treated with more aggressive surgical care and/or adjuvant treatment than their older counterparts [4], and who have higher survival rates. Furthermore, given that younger non-cancer patients (controls) tend to utilize the health care system less than older non-cancer patients, costs tend to be lower in younger control subjects, thus leading to higher net costs in younger cancer patients [4]. At the aggregate level, costs are likely higher for the four most prevalent cancer sites, such as prostate, breast, colorectal and lung, due to the higher incidence and survival [22], but also for leukemia and lymphoma, given their high lifetime costs and relatively high incidence rates [37].

Our estimates are based on data from 1997 to 2007, which were available to us at the time, and may not be reflective of more recent diffusion of newer chemotherapy agents and other changes in cancer care. This may be particularly relevant for sites, such as melanoma and prostate cancer, where the recent introduction of expensive drugs, such as ipilimumab (for melanoma), and abiraterone (for prostate cancer), have likely contributed to higher treatment costs. Given these recent innovations, it will be important for future research to examine these changes on costs.

Nonetheless, this study provides relevant insight on how costs vary across all major cancer sites and phases of care. Furthermore, our study employed rich administrative data and included a large population-based sample of *all* adults age 18+ diagnosed with cancer; most studies have included patients age 65+ only. We estimated costs for cancer sites that are not typically reported, such as multiple myeloma, and those more common among younger adults, such as thyroid and testicular cancers. These estimates are currently lacking in the literature. We used detailed costing methods and considered the majority of health services covered by the public third-party payer under a comprehensive universal health care insurance plan. We included costs for resources, such as outpatient prescription drugs and long-term care, which were not included in SEER-Medicare studies [29]. These make up a significant proportion of costs for older patients with cancer. We also made use of more rigorous matching than previous work [4], as imperfect matching can produce biased net cost estimates [38], and we used separately matched controls for the initial and terminal phases. Nonetheless, we were limited by number of variables we could match on. Given the similarity in patterns of care across the developed world, these results may be relevant to other jurisdictions that lack comprehensive population-based cancer cost estimates for all adults [39].

We were unable to provide cost estimates by cancer stage; this information was not available in the Ontario Cancer Registry during our analysis period. We were only able to capture costs for outpatient drugs covered by the public provincial drug program (patients age 65+ and special cases). As such, our cost estimates are likely an underestimate of the cost of drugs for managing treatment side effects and/or drugs for symptom management in advanced disease. Furthermore, we only estimated direct costs incurred by the public third-party payer; we did not account for other relevant costs, such as out-of-pocket or time and productivity costs. These costs are generally not readily available as they need to be collected and/or estimated through patient questionnaires. Out-of-pocket and time costs can vary by cancer site and socioeconomic status [40]. Previous work has shown that even in Ontario, a jurisdiction with public health care insurance, out-of-pocket and time costs can represent a significant burden for low-income prostate cancer patients, and have an important impact on their quality of life [41]. This should also be the focus of future research.

Finally, the estimation of lifetime costs required making some assumptions. First, we assumed all patients would be deceased 25 years after diagnosis [29]. This may be valid for most cancer sites, especially those diagnosed among older patients, but not for those typically diagnosed among younger patients, such as thyroid and testicular cancers. Nonetheless, to ensure comparability, we estimated lifetime costs in the same manner for all cancer sites. Second, we assumed no structural changes over time in technology or medical practice patterns; this is likely unrealistic but a necessary simplification in order to make use of our existing cost estimates.

## Conclusions

In conclusion, our results suggest that costs of cancer care are substantial and vary by cancer site, phase of care and time horizon of analysis. These cost estimates are valuable to decision makers to understand the economic burden of cancer care. In addition, they may be useful inputs to researchers undertaking cancer-related economic evaluations.

## Additional files

Additional file 1: Table S1. ICD-O and histology codes for cancer sites. **Table S2.** Databases^1^ and resources. **Figure S1.** Mean net costs of care by phase of care and tumour site for males (A) and females (B)*. (DOC 216 kb)
Additional file 2: Match at death. (XLSX 88 kb)
Additional file 3: Match at diagnosis. (XLSX 91 kb)

## Abbreviation

MOHLTC: Ministry of Health and Long-term Care

## Footnotes

1 The covariance of costs for cases and controls is independent, given the conditional independence of the comorbidity and socio-demographic variables after matching.

2 We have adjusted the formula as found in Yabroff et al., 2008 to address a typo.
