# Supplementary material for: Phase-specific and lifetime costs of cancer care in Ontario, Canada
Source: BMC Cancer. 2016 Oct 18;16:809. doi: 10.1186/s12885-016-2835-7 (PMC5070134; doi:10.1186/s12885-016-2835-7)
Supplement: Additional file 1: Table S1. — ICD-O and histology codes for cancer sites. Table S2. Databases1 and resources. Figure S1. Mean net costs of care by phase of care and tumour site for males (A) and females (B)*. (DOC 216 kb) [file 12885_2016_2835_MOESM1_ESM.doc]

**Supplement**

**Table S1**: ICD-O and histology codes for cancer sites

| **Cancer** | **ICD-O Code(s)** | **Inclusionary Histology Codes** | **Exclusionary Histology Codes** |
| --- | --- | --- | --- |
| Head and neck | C00.* – C06.*,  C09.* – C14.*  C30.* – C32.* | 8004, 8010, 8011, 8020, 8041, 8042, 8051, 8070–8073, 8082, 8083, 8090, 8200, 8211, 8246, 8260, 8263, 8430 | - |
| Esophagus | C15.* | 8000, 8010, 8012, 8020, 8041, 8046, 8070–8072, 8076, 8083, 8123, 8140, 8144, 8145, 8260, 8480, 8481, 8490, 8560 | - |
| Thyroid | C73.9 | 8000, 8010, 8021, 8050, 8070, 8071, 8140, 8260, 8290, 8330, 8331, 8335, 8337, 8340–8344, 8350, 8510 | - |
| Gastric | C16.* | 8000, 8010, 8020, 8070, 8071, 8140, 8142, 8144, 8145, 8210, 8211, 8240, 8246, 8255, 8260, 8263, 8480, 8481, 8490, 8560 | - |
| Colorectal | C18.0, C18.2 – C18.9  C19.9  C20.9  C21.8 | 8000, 8010, 8020, 8070, 8140, 8144, 8210, 8211, 8221, 8240, 8246, 8260, 8261, 8262, 8263, 8310, 8480, 8481, 8490, 8510 | - |
| Liver | C22.0 | 8000, 8001, 8010, 8012, 8020, 8033, 8170–8172, 8174, 8175, 8180, 8440 | - |
| Pancreas | C25.* | 8000, 8001, 8010, 8012, 8020, 8041, 8046, 8140, 8150, 8240, 8246, 8260, 8440, 8470, 8480, 8481, 8490, 8500, 8550, 8560 | - |
| Lung | C33.9  C34.* | 8000, 8010, 8012, 8020, 8040, 8042, 8046, 8070–8072, 8140, 8240, 8246, 8250, 8255, 8260, 8480, 8481, 8550, 8560 | - |
| Female breast | C50.* (female) | 8000, 8010, 8050, 8140, 8211, 8480, 8500, 8501, 8503, 8504, 8510, 8520–8524, 8530, 8541, 8543, 8575 | - |
| Corpus uteri | C54.* | 8000, 8010, 8020, 8050, 8070, 8072, 8140, 8210, 8260, 8263, 8310, 8323, 8380, 8441, 8460, 8461, 8480, 8481, 8560, 8570 | - |
| Cervix | C53.* | 8010, 8041, 8052, 8070–8072, 8076, 8100, 8140, 8246, 8260, 8263, 8310, 8380, 8384, 8460, 8480, 8481, 8560, 8570 | - |
| Ovary | C56.9 | 8000, 8010, 8020, 8070, 8140, 8260, 8310, 8323, 8380, 8441, 8442, 8460–8462, 8470, 8472, 8480, 8620, 9060, 9080 | - |
| Testis | C62.* | 8000, 8001, 8010, 8140, 8650, 8940, 9061, 9062–9065, 9070, 9071, 9080, 9081, 9083, 9085, 9100, 9101, 9364 | - |
| Prostate | C61.9 | 8000, 8010, 8041, 8046, 8070, 8130, 8140, 8145, 8201, 8255, 8260, 8310, 8340, 8341, 8380, 8480, 8481, 8490, 8500, 8550 | - |
| Bladder | C67.* | 8000, 8001, 8010, 8020, 8033, 8041, 8050, 8070, 8071, 8120, 8122, 8130, 8191, 8140, 8246, 8260, 8310, 8480, 8481, 8490 | - |
| Kidney | C64.9  C65.9 | 8000, 8010, 8012, 8050, 8070, 8120, 8130, 8140, 8255, 8260, 8270, 8290, 8310, 8312, 8316–8320, 8323 | - |
| Brain | C71.* | 8000, 8140, 9380, 9382, 9391, 9392, 9400, 9401, 9411, 9420, 9421, 9424, 9430, 9440–9442, 9450, 9451, 9470, 9573 | - |
| Melanoma | C44.* | 8720 – 8790  (with or without ICD-10 code C44.*) | - |
| Lymphoma | C77.* | 9650–9667, 9590–9596, 9670, 9671, 9673, 9675, 9678–9680, 9684, 9687, 9689–9691, 9695, 9698–9702, 9705, 9708, 9709, 9714–9719, 9727–9729, 9823, 9827–9829 | - |
| Multiple myeloma | - | 9731, 9732, 9734 | - |
| Leukemia | C42.* | 9733, 9742, 9800, 9801, 9805, 9820, 9823, 9826, 9827, 9831–9834, 9835–9837, 9840, 9860, 9861, 9863, 9866, 9867, 9870–9876, 9891, 9895, 9897, 9910, 9920, 9930, 9931, 9940, 9945, 9946, 9948, 9963, 9964 | - |
| Other tumour sites | C07.* – C08.*  C17.*  C18.1  C22.1  C24.*  C26.0, C26.8 – C26.9  C37.9  C38.* - C41.*  C42.2, C42.3  C47.* - C49.*  C50.* (male)  C51.*  C52.9  C57.*  C58.9  C60.*  C63.*  C68.* – C70.*  C72.*  C74.* – C75.*  C80.9 |  | 9590 – 9989 Leukemia, lymphoma and multiple myeloma |

**Legend:** ICD-O – International Classification of Diseases-Oncology

**Table S2**: Databases^1^ and resources

| **Database** | **Resources** |
| --- | --- |
| New Drug Funding Program | chemotherapy drugs |
| Activity Level Reporting System | radiation therapy |
| Ontario Health Insurance Plan Claims History Database | diagnostic tests, physician services, chemotherapy visits and emergency department visits pre-2002 |
| Ontario Drug Benefit plan data | outpatient prescription drugs for patients aged >65, oral anti-neoplastic drugs, long-term care indicator |
| CIHI- Discharge Abstract Database | inpatient hospitalizations, same-day surgery pre-2002 |
| CIHI-National Ambulatory Care Reporting System | emergency department visits post-2002; same-day surgery post-2002 |
| Continuing Care Reporting System | stays in complex continuing care facilities |
| Ontario Home Care Administrative System | home care pre-April 2005 |
| Home Care Database | home care post-April 2005 |

^1^ All databases were available at the Institute for Clinical Evaluative Sciences, Toronto Ontario, Canada, with the exception of the New Drug Funding Program and Activity Level Reporting System which were obtained from Cancer Care Ontario.

**Legend:** CIHI – Canadian Institute for Health Information

**Figure S1**: Mean net costs of care by phase of care and tumour site for males (A) and females (B)*

A


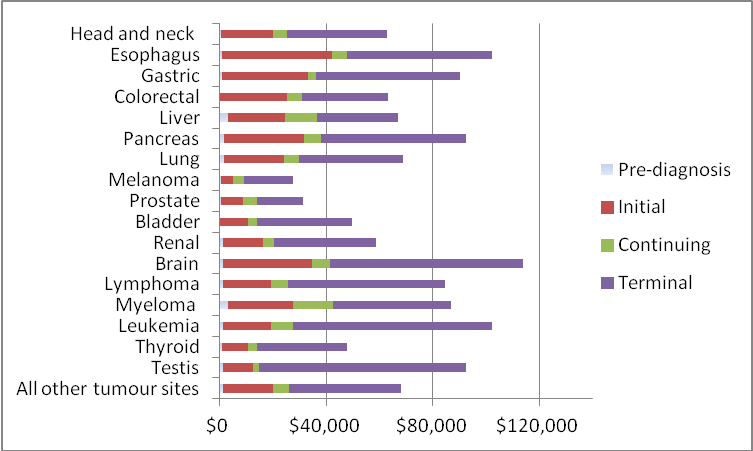


B


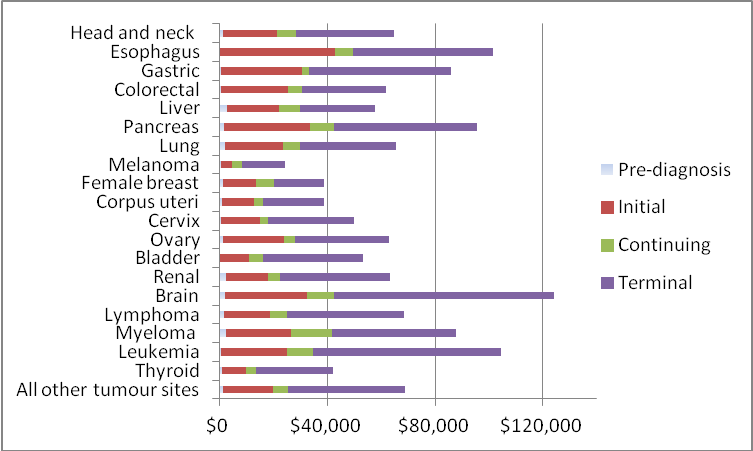


* The initial phase of care is the first 6 months following diagnosis, the terminal phase is the final 12 months of life, and the continuing phase is all the months between the initial and last year of life phases. Net costs in the continuing phase of care are an annual estimate. Net costs in the last year of life combine the cost for cancer patients dying of cancer and those dying of other causes. All estimates are in 2009 dollars.

† All other tumour sites includes salivary gland, small intestine, appendix, intrahepatic bile duct, gallbladder and extrahepatic bile ducts, unspecified digestive organs, pleura, thymus, heart, mediastinum, other respiratory organs, bones and joints, reticulo-endothelial, spleen, connective tissue/nerves, retroperitoneum and peritoneum, soft tissue, breast (male only), labia and clitoris, vulva, vagina, other female genitals, placenta, penis, epididymis, spermatic cord, scrotum, other and unspecified male genitals, other urinary organs, ureter, eye, orbit and lacrimal gland, eye (unspecified), cerebral and spinal meninges, meninges NOS, spinal cord, cranial nerves, other nervous system, adrenal glands, parathyroid gland, pituitary gland, craniopharyngeal duct, pineal gland, other endocrine glands and miscellaneous (ill-defined and unknown organs).

Data sources: Cancer Care Ontario and administrative health data housed at the Institute for Clinical Evaluative Sciences.
